# Entanglement distribution in lossy quantum networks

**DOI:** 10.1038/s41598-025-14226-2

**Published:** 2025-08-14

**Authors:** Leonardo Oleynik, Junaid ur Rehman, Seid Koudia, Symeon Chatzinotas

**Affiliations:** 1 Interdisciplinary Centre for Security, Reliability and Trust (SnT), University of Luxembourg, Luxembourg, L-1855 Luxembourg; 2 Department of Electrical Engineering, and the Center for Intelligent Secure Systems, King Fahd University of Petroleum and Minerals (KFUPM), Dhahran, 31261 Saudi Arabia

**Keywords:** Entanglement distribution, Lossy quantum networks, W states, Quantum information, Qubits

## Abstract

Entanglement distribution is essential for unlocking the potential of distributed quantum information processing. We consider an N-partite network where entanglement is distributed via a central source over lossy channels, and network participants cooperate to establish entanglement between any two chosen parties under local operations and classical communication (LOCC). We develop a general mathematical framework to assess the average bipartite entanglement shared in a lossy distribution, and introduce a tractable lower bound by optimizing over a subset of single-parameter LOCC transformations. Our results show that probabilistically extracting Bell pairs from W states is more advantageous than deterministically extracting them from GHZ-like states in lossy networks, with this advantage increasing with network size. We further extend our analysis analytically, proving that W states remain more effective in large-scale networks. These findings offer valuable insights into the practical deployment of near-term networks, and corroborate a trade-off relationship between the success conversion probability of entanglement distribution protocols and their robustness to loss.

## Introduction

Quantum entanglement is fundamental for realizing the potential of distributed quantum information processing (DQIP). In this context, entanglement can be usefully pictured as a resource that is harnessed by physically separated parties, constrained by local operations and classical communication (LOCC), to perform various informational tasks. In such LOCC transformations, each party can measure their part of the system and broadcast the outcomes through a classical channel. This broadcasted information subsequently informs updates in the measurements of other parties. Understanding the capabilities and limitations of such operations is crucial, as many quantum information tasks, including teleportation, superdense coding^1^, one-way quantum computation, quantum secure direct communication^2,3^, quantum conference key agreement, and entanglement distribution, rely on the LOCC paradigm.

*Random*-party entanglement distillation (RED) is a critical problem in DQIP. It involves investigating protocols to transform multipartite entangled states into a Bell pair shared among *unspecified* parties. The advantages of random-party over specified-party entanglement distillation protocols were first highlighted in^4–6^. In particular, it was shown that a W state can be reliably converted into a Bell pair via a RED protocol with a probability of success asymptotically reaching the unity for infinitely many rounds of LOCC operations^4^ — and, contrary to multiple-copies settings^7–9^, such protocol does not resort to quantum purification as it relies on a single copy of a W state. Although initially promising, subsequent findings demonstrated that LOCC operations cannot achieve this limit^10^, as they belong to a class of operations that is not topologically closed^11,12^. These results have revealed the major role of LOCC round complexity analysis in RED protocols, a topic only recently scrutinized in the specific case of W states^13^. Altogether, the study of RED protocols has many subtleties and open questions.

W and Greenberger-Horne-Zeilinger (GHZ) states represent two distinct, nonequivalent entanglement classes for three-qubit systems^14,15^. As claimed in^16^, their distinction lies in their entanglement structure: Although any bipartition of a GHZ state has maximal entanglement (exactly one ebit), the bipartite entanglement content of a W state is strictly less than one ebit. This structure results in two complementary aspects: while GHZ states can be *deterministically* converted into Bell pairs (e.g., with a single qubit measurement in the Pauli-*X* basis) but are sensitive to loss (all the entanglement is gone if any of the qubits are lost); W states can only be *probabilistically* converted into Bell pairs, yet are robust to loss (some entanglement can be retrieved even if any of the qubits are lost). In this sense, for three-qubit systems, there is a clear tradeoff relationship between the success probability of entanglement conversion protocols for a given resource and the resource’s robustness to losses. For instance, a deterministic conversion comes at the expense of loss sensitivity, making each class preferable depending on the distributed information-processing scenario.

Although entanglement distillation has been widely studied in both random and specified scenarios, few works have addressed these aspects in the context of near-term quantum networks^17–20^. Motivated by the tradeoff relationship between the success probability of entanglement conversion protocols for a given resource and the resource’s robustness to losses present in three-partite entangled states, we developed a theoretical framework to assess bipartite entanglement conversion of arbitrary resources in a lossy network and employed it to compare W and GHZ-like states’ performance in different network settings. This work examines the performance of single-copy, RED protocols in an *N*-partite centralized lossy network. Our primary contributions include:

- Developing a comprehensive theoretical framework to compute the average bipartite entanglement shared among a pair of parties within an *N*-partite centralized lossy network;
- Assessing single-parameter LOCC entanglement conversion performance for W and GHZ-like states by developing computationally efficient lower bounds for the figure of merit;
- Demonstrating W state’s advantage over GHZ-like states numerically (and analytically for particular cases), corroborating the extension of a tradeoff relationship between the success probability of entanglement conversion protocols for a given resource and the resource’s robustness to losses to multi-party states.

The remainder of this paper is organized as follows. In Section 1, we define the general problem, mathematical model, and notation, as well as our figure of merit and benchmark. In Section 2, we introduce a tractable lower bound for our figure of merit and its simplifications for single-round, single-parameter (SP) LOCC transformations, applying it in Section 3 to compare the performance of W and GHZ-like states in lossy entanglement distribution. We conclude our discussion and provide possible future directions in Section 4.

## Problem statement: entanglement distribution in a lossy network

We consider an *N*-partite network that is served by a centralized source that generates and then distributes an *N*-qubit state (resource) $\psi$ to the network participants. The links from the source to each node are assumed to be lossy, i.e., a particle generated at the source gets lost in the link with probability $\epsilon$. Losses in all links are considered to be independent and identically distributed. The participants cooperate by measuring the received particles to generate entanglement between any two participants *A* and *B*. Each node can perform $r_i\ge 1$ rounds of measurement on its received particle and broadcast the outcomes of each round to the network via a classical broadcast channel, i.e., the parties are constrained by LOCCs. Given this entanglement distribution scenario, we want to:

1. Evaluate the amount of entanglement shared on average between two network participants as a function of $\epsilon$, for a given parameter region $(N,r_i)$ and a resource state $\psi$;
2. Compare W and GHZ-like states’ performance over different parameter regions, including asymptotic regimes (e.g., $N\rightarrow \infty$).

Next, we formalize the above concepts and goals.

**Fig. 1:**
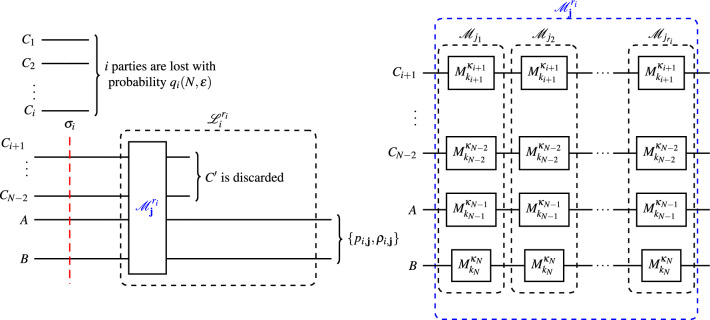
On the left, entanglement distribution, and LOCC processing in a lossy network. On the right, the sequence of $r_i$ global measurements $\mathscr {M}_{j_x}$, namely $\mathscr {M}_\textbf{j}^{r_i}$, is detailed.

### Mathematical model and notations

We arbitrarily label the participants of interest by *A* and *B* and the helper participants by *C* with an index, i.e., $C_1,C_2,...,C_i,...,C_{N-2}$ as depicted in Fig. 1. Labeling is arbitrary because in a RED protocol success is deemed whenever some entangled bipartite state (not necessarily the maximally entangled one) is obtained between *any* two parties^5,12,13^. In the distribution phase, any of the *N* particles can be lost, but if more than $N-2$ are lost, nothing can be done locally to increase the entanglement. Then, the probability that $i\in \left\{ 0, 1, \cdots , N-2\right\}$ particles are lost is

1 $$\begin{aligned} q_i\left( N, \epsilon \right) =\left( {\begin{array}{c}N-2\\ i\end{array}}\right) \epsilon ^i (1-\epsilon )^{N-2-i}. \end{aligned}$$

When exactly *i* particles are lost, the overall state of the system is

2 $$\begin{aligned} \sigma _i^N:= \text {Tr}_{C_1, C_2,...C_i} \vert {\psi }\rangle \langle {\psi }\vert , \end{aligned}$$

or simply $\sigma _i$ when the $\psi$’s dimension can be inferred from the context. For the particular case of W and GHZ states, the above equation simplifies to

3 $$\begin{aligned} \sigma _i^N = \frac{1}{N} \left[ {\vert {0}\rangle \langle {0}\vert ^{\otimes N-i} + (N-1) \sigma _{i-1}^{N-1}}\right] , \end{aligned}$$

and

4 $$\begin{aligned} \sigma _i^N = \frac{I_{N-i}}{N-i} \equiv \pi _{N-i} \end{aligned}$$

respectively, with $\sigma _0^N = \vert {\psi }\rangle \langle {\psi }\vert$. These equations explicitly show the loss-robustness (-sensitivity) of W (GHZ) states: while losing any number of particles from a GHZ state results in a maximally mixed state (4), with no bipartite entanglement; losing up to $N-2$ particles from a W state results in a noisy EPR pair, which can be seen by applying (3) recursively. The received states and their corresponding probabilities form an ensemble

5 $$\begin{aligned} \psi \rightarrow \{ q_i, \sigma _i \}. \end{aligned}$$

After the distribution phase, all the remaining parties, $C_{i+1}, C_{i+2},..., C_{N-2}=C'$, *A*, and *B*, take turns measuring their local systems and broadcasting the results. Since each party holds a qubit, local measurements can be described by the $2\times 2$ Kraus operators $\{ M_{k}^{\kappa } \}_k$. We can assume, without loss of generality^13^, that each $M_{k}^{\kappa }$ is in the upper triangular form

6 $$\begin{aligned} M_{k}^{\kappa } = \begin{pmatrix} \sqrt{a_k} & b_k \\ 0 & \sqrt{c_k} \end{pmatrix}, \end{aligned}$$

where ${\kappa }=(a_k,b_k,c_k)$, $a_k,c_k\le 0$, and the completion relation implies that $\sum _k a_k =1$ and $\sum _k c_k \ge 1$.

Each received state $\sigma _i$ is processed by an $r_i$-*round LOCC transformation* $\mathscr {L}_i^{r_i}$ that converts it into a state $\rho _{i,\textbf{j}}$ with probability $p_{i,\textbf{j}}$. In other words, an $r_i$-*round LOCC transformation* can be viewed as a map

7 $$\begin{aligned} \mathscr {L}_i^{r_i}: \sigma _i \rightarrow \{ p_{i,\textbf{j}}, \rho _{i,\textbf{j}} \}, \end{aligned}$$

where

8 $$\begin{aligned} p_{i,\textbf{j}}&= \text {Tr} \left( {\mathscr {M}_\textbf{j}^{r_i} }\right) ^\dagger \mathscr {M}_\textbf{j}^{r_i} \sigma _i, \end{aligned}$$

9 $$\begin{aligned} \rho _{i,\textbf{j}}&= \frac{ \text {Tr}_{C'} \mathscr {M}_\textbf{j}^{r_i} \sigma _i\left( {\mathscr {M}_\textbf{j}^{r_i} }\right) ^\dagger }{p_{i,\textbf{j}}}. \end{aligned}$$

In the above expression, $\mathscr {M}_\textbf{j}^{r_i}$ represents a sequence of $r_i$ global measurements given by the following composition rule

10 $$\begin{aligned} \left\{ \mathscr {M}_\textbf{j}^{r_i} = \bigcirc _{x=1}^{r_i} \mathscr {M}_{j_x} \right\} _{\textbf{j}=j_{r_i},j_{r_i-1},...,j_1} \end{aligned}$$

wherein every $\mathscr {M}_{ j_{x}}$ is expressed as the tensor product of LOCCs over the remaining $N-i$ subspaces, i.e.,

11 $$\begin{aligned} \left\{ \mathscr {M}_{ j_{x}} = \otimes _{m=1}^{N-i} M_{k_{m}}^{{\kappa }_{m}} \right\} _{{j_x = k_i+1,k_i+2,\ldots ,k_N}}. \end{aligned}$$

in which $M_{k_{m}}^{{\kappa }_{m}}$ has the form of (6), parameterized by ${\kappa }_m=(a_{k_m},b_{k_m},c_{k_m})$. It is worth noting that, in general, the LOCCs performed in each node differ among the nodes ${\kappa }_m \ne {\kappa }_{m'}$ and from round to round $\mathscr {M}_{ j_{x}} \ne \mathscr {M}_{ j_{x'}}$. Likewise, each node can perform its LOCCs a different number of times $r_i \ne r_{i'}$. Later, in order to simplify our problem, we will consider a particular class of transformations where the LOCCs are identical in every round, identical for every system and each node is constrained by the same amount of rounds.

We are interested in quantifying the amount of bipartite entanglement shared on average among *A* and *B* after $r_i$ rounds of LOCC transformations, i.e., the entanglement content of the reduced state $\rho _{i,\textbf{j}}$. Since system labeling is arbitrary for RED protocols, we can always choose *AB* as the bipartition with the maximum amount of entanglement and $C'$ as the remaining partitions. This implies in *equivalent states*, i.e., reduced states with the same amount of bipartite entanglement. Next, we present the figure of merit that takes this into account as well as its benchmark.

### Figure of merit and benchmark

We propose *the average bipartite entanglement optimally shared among the target parties through a lossy network in *$\textbf{r}= (r_0, r_1,..., r_i,...,r_{N-2})$ *rounds* as the figure of merit, defined as follows

12 $$\begin{aligned} \langle {E}\rangle _{\textbf{r},\psi }(\epsilon ) := \sum _i q_i(N,\epsilon ) \bar{E}^{*}_{{r_i},{\sigma _i}} , \end{aligned}$$

stop where

13 $$\begin{aligned} \bar{E}^{*}_{{r_i},{\sigma _i}} := \sup _{ \mathscr {L}_i^{r_i} } \sum _{\textbf{j}} p_{i,\textbf{j}} E(\rho _{i,\textbf{j}}), \end{aligned}$$

and *E* is some bipartite entanglement measure, e.g., the entanglement of formation or concurrence^21^. We will adopt the latter in our numerical evaluations.

The definitions above are averages of the shared bipartite entanglement over the lossy distribution (5) and the probabilistic conversion (7) distributions. More specifically, whereas (13) is the average bipartite entanglement optimally achieved given a received state $\sigma _i$ via $\mathscr {L}_i^{r_i}$ protocols; (12) is the average of (13) over the ensemble of received states. Therefore, our definitions fully consider the statistical nature of the problem.

A similar definition to (13) is presented in^13^ for a different entanglement distribution context. Applied to our distribution scenario, this definition could be written as $\sup _{r_i} \bar{E}^{*}_{{r_i},{\sigma _i}}$ and interpreted as the optimal average bipartite entanglement given any LOCC protocol, which includes protocols with an unbounded number of rounds. We adopted (13) to avoid unbounded LOCC protocols and to distinguish the effect of the number of rounds in the figure of merit.

According to^22^, for finite-copy entanglement distillation, an entanglement *measure* is any nonnegative function *E* which is monotonically nonincreasing under LOCC transformations. Despite the lack of consensus on the necessary conditions that an entanglement measure must satisfy, monotonicity is often claimed as the essential property^22^. In a quick inspection, we observe that (13) is an entanglement measure by definition, while (12) is also an entanglement measure as it represents the average of (13).

We benchmark W states against two-centered GHZ graph states. As detailed in the Appendix, two-centered GHZ graph states have the property of being loss-robust. More precisely, it has been shown^23^ that if multiple qubits adjacent to the same root are lost, the remaining state is always a GHZ state, and if qubits adjacent to both roots or the roots themselves are lost, the remaining state is fully separable. Such an entanglement structure simplifies our analysis, as after the distribution phase the received state is either a GHZ state, which can be deterministically converted to Bell pairs in a single round with a single measurement, or is fully separable, having no distillable entanglement whatsoever. In both cases, (13) is trivially and exactly computed — $\bar{E}^{*}_{{r_0},{\sigma _0}} = 1$ if none of the qubits is lost and $\bar{E}^{*}_{{r_i},{\sigma _i}} = 0$ if any qubit is lost.

## Methodology

In this section, we establish a tractable lower bound for the figure of merit by optimizing (12) over the subset of single-parameter LOCC (SP-LOCC) transformations. We then analyze the properties of single-round, SP-LOCC transformations to derive explicit expressions for (9) and enhance computational efficiency in evaluating the lower bound.

### Lower bound

In the general formulation of the problem, the local operations performed are different from one node to the other and have the form (6) whose parameters $a_k, b_k, c_k$ can change in every round of the protocol, as emphasized in 1.1. For simplicity, we will restrict ourselves to the family of *SP-LOCC transformations* $\mathscr {L}_i^{r,\kappa }$, where the measurements are

1. identical for every system, i.e., ${\kappa }_m = {\kappa }_{m'} \,\forall \, m,m' \in [1,N-i]$
2. identical in every round, i.e., $\mathscr {M}_{ j_{x}} = \mathscr {M}_{j_{x'}} \,\forall \, x$ and $x' \in [0,r_i]$
3. single parameterized ${\kappa }_m =\kappa$, i.e., the Kraus operators have the simplified form
  14 $$\begin{aligned} M_0^\kappa =\begin{pmatrix} \sqrt{1-\kappa } & 0 \\ 0 & 1 \end{pmatrix}, \quad \quad M_1^\kappa =\begin{pmatrix} \sqrt{\kappa } & 0 \\ 0 & 0 \end{pmatrix}, \end{aligned}$$
  where $\kappa \in [0,1]$ as proposed in^4^.

Moreover, we assume the same number of rounds for every received state, i.e., $r_i=r \, \forall \, i$. These assumptions allow us to simplify the set of Kraus operators (10) and (11) as follows

15 $$\begin{aligned} \left\{ \mathscr {M}_\textbf{j}^r = \bigcirc _{x=1}^{r} \mathscr {M}_{j_x}\right\} _{\textbf{j}=j_r,j_{r-1},...,j_1} \end{aligned}$$

with

16 $$\begin{aligned} \left\{ \mathscr {M}_{j_x} = \otimes _{m=1}^{N-i} M_{k_m}^{\kappa }\right\} _{{j_x = k_i+1,k_i+2,\ldots ,k_N}}. \end{aligned}$$

These assumptions are the same as presented in^4^ to show the asymptotic optimality of converting a three-party *W* state in a Bell pair. Here we extend them to any $\sigma _i$ state, which includes mixed states $\sigma _{i\ne 0}$.

By considering only the family of *SP-LOCC transformations* $\mathscr {L}_i^{r,\kappa }$, we define the following lower bound

17 $$\begin{aligned} \langle {{\hat{E}}\rangle _{r,\psi }(\epsilon )}:=\sum _i q_i(N,\epsilon ) \sup _{\kappa } \sum _{\textbf{j}} p_{i,\textbf{j}} E(\rho _{i,\textbf{j}}), \end{aligned}$$

which is trivially upper bounded by (12) as the optimization runs over the subset of transformations. In the above, $\kappa$ is a function of *r*, since bigger and smaller protocols have different optimal sets of LOCCs.

### SP-LOCC transformations applied to W states

Finite-state Markovian chains (FSMCs) set the mathematical framework to compute $p_{i,\textbf{j}}$ and to keep track of $\rho _{i,\textbf{j}}$ in a *r*-round SP-LOCC transformations. More precisely, by associating every set $\{\rho _{i,\textbf{j}}^r \}_\textbf{j}$ to the sampled values of a random variable $X_r$ for every $r\ge 0$, the process $\{X_r\}_{r\ge 0}$ can be interpreted as an FSMC process — i.e., its Markovian state space $\mathscr {X}$ is a finite set, and its evolution only depends on the previous time step (see also the Appendix for the complete proof).

**Fig. 2:**
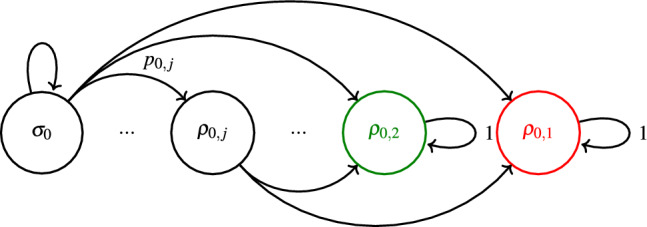
Markovian chain representation of a lossless *r*-round LOCC protocol $\mathscr {L}_0^{r}$. Nodes and arrows represent the quantum states of (18), and their corresponding probabilities, respectively. Further, end nodes (that terminate the protocol once reached) are depicted in green and red, representing, respectively, Bell and separable states.

The corresponding Markovian state space and transitioning probabilities of a lossless *r*-round SP-LOCC transformations $\mathscr {L}_0^r$ are depicted in Fig. 2 with states given by

18 $$\begin{aligned} \rho _{0,j}= {\left\{ \begin{array}{ll} W_j & \text {if}\quad j \ge 3 \\ \phi & \text {if}\quad j =2 \\ \left| {0}\right\rangle ^{\otimes N} & \text {if}\quad j = 1 , \end{array}\right. } \end{aligned}$$

where *j* is the number of 0s in the string $\textbf{j}={j_x = k_i+1,k_i+2,\ldots ,k_N}$, $W_j$ is a *j*-qubit W state and $\phi$ is an EPR pair. This protocol is an adaptation of Fortescue and Lo’s protocol^4^ to a *N*-qubit W state (see also the Appendix). For the general case $i>0$ the Markovian state space increases polynomially with the number of rounds and number of nodes *N* and full pictorial representation is not possible. In the following, we discuss the particular case of single-round SP-LOCC transformations. To avoid notation clutter, we will omit $r=1$ in the following sections.

#### Single-round SP-LOCC transformations applied to W states

Given the symmetry of $\sigma _i=\text {Tr}_{C'} W$, the form of (14) and the arbitrary discardment of $C'$, there will be reduced states with the same amount of entanglement. These so-named equivalent states lead to redundancies in the evaluation of (17), the same term in the second summation is computed and optimized repeatedly, which naturally slows the computation. To eliminate these redundancies and speed up the numerical computations, as well as to obtain simpler analytical expressions, we focus on the subset of non-equivalent states and their corresponding probabilities.

**Fig. 3:**
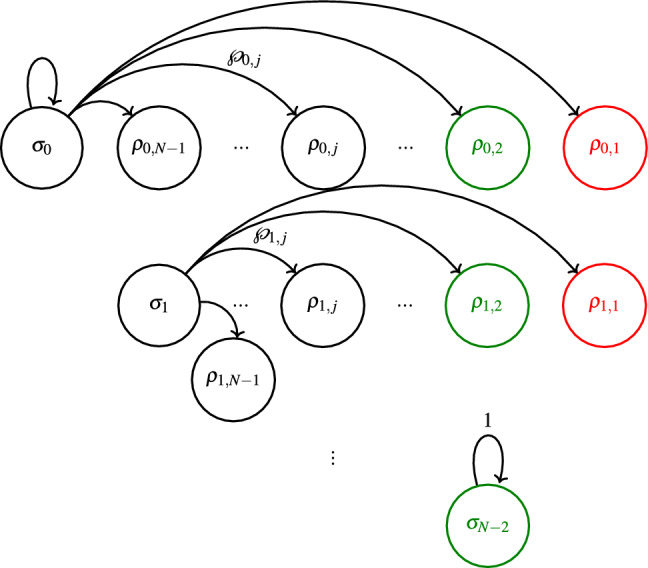
Markovian chain representation of a single-round LOCC protocol. Nodes and arrows represent the non-equivalent ensemble given by (20). Further, end nodes (that terminate the protocol once reached) are depicted in green and red, representing, respectively, Bell and separable states. Note that every line corresponds to an independent Markov chain, where the first one represents the lossless single-round LOCC, a special case of Fig. 2.

Therefore, we define the following non-equivallent ensemble $\{\wp _{i,j}, \rho _{i,j} \}$, where $\wp _{i,j}$ is the probability of obtaining any equivalent state $\rho _{i,j}$. This restriction allows us to find the following explicit expressions

19 $$\begin{aligned} \wp _{i,j}&= \left( {\begin{array}{c}N-i\\ j\end{array}}\right) \frac{j}{N-i} \kappa ^{N-i-j} (1-\kappa )^{j-1} \end{aligned}$$

20 $$\begin{aligned} \rho _{i,j}&= \left( {\begin{array}{c}N-i\\ j\end{array}}\right) \frac{\text {Tr}_{C'} \mathscr {M}_{j} \sigma _i\mathscr {M}_{j} }{\wp _{i,j}} \end{aligned}$$

where *j* is the number of 0s in the string $\textbf{j}=j_1=k_1,k_2,...,k_m,...,k_{N-i}$. The first simplification comes from observing that permutations of $k_m$ in $\textbf{j}$ do not alter the number of 0s and therefore correspond to the same state. The second is simply (9) expressed in terms of $\wp _{i,j}$. These states and probabilities can be mapped to a single-trial FSMC as depicted in Fig.  3.

## Application

In this section, we use the previously defined lower bound (17) to compare the performance of W and GHZ-like states in our lossy entanglement distribution scenario 1. Our results demonstrate that, in a lossy network, extracting Bell pairs from W states is more advantageous than from GHZ-like states, even though the former is only achievable probabilistically, whereas the latter can be done deterministically. Furthermore, we analytically extend these findings in the Appendix, proving that W states serve as more effective resources in large networks.

### W states’ advantage in lossy networks

**Fig. 4:**
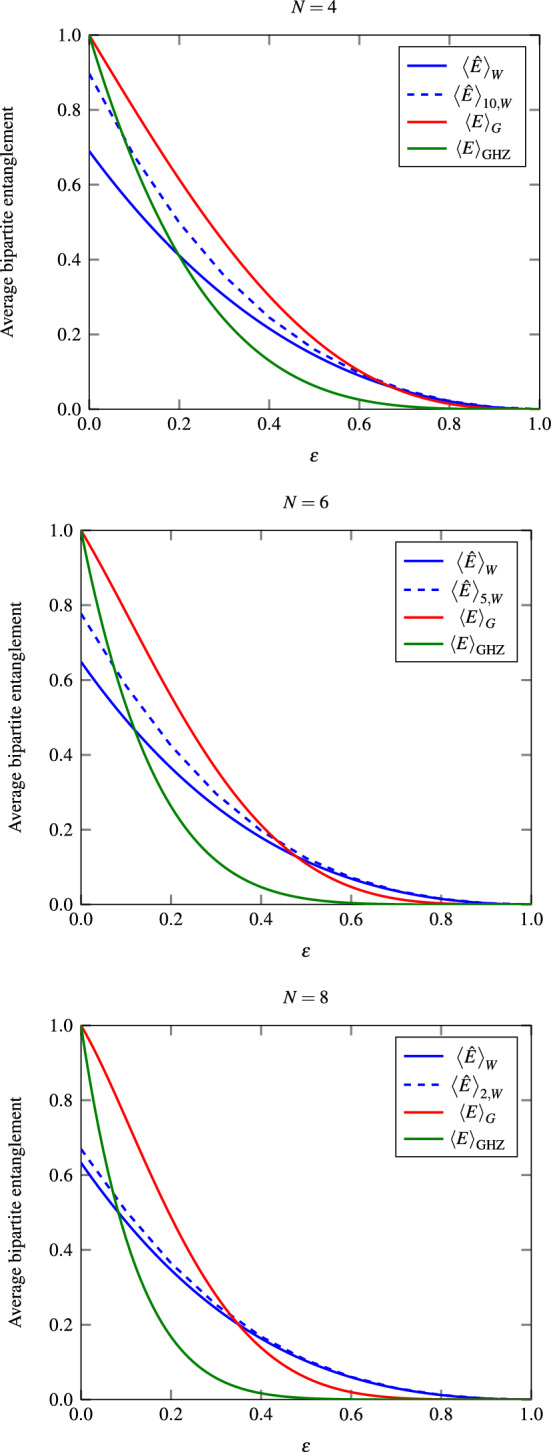
The average bipartite entanglement shared between the target parties in a lossy network, considering single-round (multiple-rounds) SP-LOCC transformations of W, GHZ and two-centered GHZ graph states as a function of the loss probability $\epsilon$ in solid (dashed) blue, red and green. From top to bottom, these quantities are depicted for a network of size 4, 6 and 8. The intersection point common to the blue and red lines defines the *lossy threshold* over which bipartite conversion of W states outperforms GHZ-like states.

We observed that probabilistically extracting Bell pairs from W states is more advantageous than doing it deterministically from GHZ-like states in lossy networks. In Fig. 4 we compare our figure of merit for W and GHZ-like states for different network sizes. It is clear that above a certain threshold in loss (when the curves intersect each other) more bipartite entanglement can be obtained on average from W than from GHZ-like states in single-round transformations, showcasing some advantage in using W states as initial resources. Moreover, such an advantage considerably increases when a higher number of rounds are allowed, as can be noticed when comparing the single- with multiple-round LOCC transformation depicted in solid and dashed lines in Fig. 4 respectively. We must stress that since we are comparing a lower bound of the average bipartite entanglement (17) extracted from the W states against the exact average bipartite entanglement extracted from GHZ-like states, the real threshold must be even lower than the one depicted in the plots. The plots also indicate that this threshold decreases as the network size increases, as observed for network sizes of $N=4,6,8$.

#### W states’ advantage in large lossy networks

The numerical examples of Fig. 4 indicate an inverse proportionality between the network size and the value of the threshold, as the network size increases from 4 to 8 the threshold decreases (from approximately .2 to .1 when comparing the W and GHZ states, for example). As we formally prove in Appendix this trend persists for any *N*, leading to the conclusion that, for large lossy networks ($N\rightarrow \infty$), extracting Bell pairs from W states is always more effective, according to our figure of merit, than from GHZ-like states. As detailed in the Appendix, the proof derives from the *loss-robustness of W states*^15^ and the inexistence of deterministic W-to-EPR state conversions^10^.

## Conclusion

In this work, we explore RED in lossy networks, focusing on whether the loss robustness of the W states outweighs the deterministic conversion of GHZ-like states into Bell pairs. We considered an *N*-partite network where entanglement is distributed through a central source over lossy channels, and network participants cooperate to establish entanglement between any two chosen parties. To analyze this scenario, we introduced a tractable lower bound for the expected shared entanglement by optimizing our figure of merit (12) over the subset of SP-LOCC transformations. By leveraging the properties of single-round SP-LOCC, we eliminated redundancies in the bound evaluation, improving general computational efficiency, and derived explicit expressions for (9).

Our results demonstrate that probabilistically extracting Bell pairs from W states is more advantageous than doing it deterministically from GHZ-like states in lossy networks. We further extended our analysis analytically, proving that W states remain more effective in large-scale networks. This has direct implications for designing optimal entanglement distribution policies — e.g., while GHZ-like states are preferable in small, low-loss networks, W states emerge as better options in large and high-loss settings. These findings provide valuable insights into the practical deployment of lossy quantum networks, highlighting the fundamental trade-offs between probabilistic and deterministic entanglement distribution protocols.

Future endeavors include finding better bounds for the figure of merit by considering the general optimization problem in (12), whose LOCC measurements are multi-parameterized and can be adjusted from round to round. Alternatively, diversity and multiplexing schemes^24,25^ could be exploited to improve RED, as entanglement distribution over a centralized QN can be framed as a multi-mode communication system^26^.

## Supplementary Information

Supplementary Information.

## Data Availability

The related data is available upon reasonable request by contacting the first author, Leonardo Oleynik.
