# Supplementary material for: Entanglement distribution in lossy quantum networks
Source: Sci Rep. 2025 Aug 14;15:29778. doi: 10.1038/s41598-025-14226-2 (PMC12350926; doi:10.1038/s41598-025-14226-2)
Supplement: Supplementary file 1 — Supplementary Information. [file 41598_2025_14226_MOESM1_ESM.pdf]

Entanglement Distribution in Lossy Quantum Networks: Supplementary Material

Leonardo Oleynik[1,*,+], Junaid ur Rehman[2,+], Seid Koudia[1,+], and Symeon Chatzinotas[1]

[1]Interdisciplinary Centre for Security, Reliability and Trust (SnT) – University of Luxembourg, L-1855 Luxembourg
[2]Department of Electrical Engineering, and the Center for Intelligent Secure Systems, King Fahd University of Petroleum and Minerals (KFUPM), Dhahran 31261, Saudi Arabia
[*]leonardo.oleynik@uni.lu
[+]these authors contributed equally to this work

ABSTRACT

In this Supplementary Material, we begin by motivating and presenting the definition of graph states, including the ones utilized in the paper. We then formalize the concept of resource advantage and demonstrate its use by comparing W and GHZ states. Following this, we introduce the FSMC framework and provide proof that SP-LOCC transformations can be modeled by it. Then, we introduce Fortescue and Lo's entanglement distillation protocol, highlighting the similarities and differences with our protocol. Finally, we present analytical expressions for our figure of merit.

1 Graph states

A graph is defined as a collection of vertices and a rule describing how they are connected by edges. They are often represented pictorially as points (the vertices) on a plane connected by arcs (the edges). Formally, a finite and undirected graph is defined by the pair

$$G = (V, E), \tag{1}$$

where $V = \{1, ..., N\}$ is the set of edges and $E \subset [V]^2$ is the set of edges and every element of E is a subset of V with two elements[1]. In the following, we define graph states by providing physical meaning to vertices and edges — i.e., we seek motivation for the concept of graph states in interaction patterns between quantum systems (alternatively, simple graphs can be associated to quantum states in terms of their stabilizer, the stabilizer formalism). The content of this section is based on[1,2].

1.1 Definition: interaction pattern

In the interaction pattern description, graph states are defined by providing physical meaning to vertices and edges. Specifically, vertices are associated with particles, whereas edges describe how those particles interact. For the particular case of qubits, a graph state can be regarded as a two-step procedure where qubits are prepared in some initial pure state $|\psi\rangle$ and are coupled according to the underlying interaction pattern given by the edges of G. Formally, for each edge $\{a, b\} \in E$, connecting qubits a and b, a local two-particle unitary $U_{ab} = e^{-i\phi_{ab}H_{ab}}$, where ϕ_{ab} and H_{ab} denote the coupling strength and the interaction Hamiltonian, respectively. To comply with the structure of a *simple and undirected* graph G, these unitaries must satisfy the following constraints:

1. they must commute, i.e.,

$$[U_{ab}, U_{bc}] = 0 \quad \forall a, b, c \in V; \tag{2}$$

2. they must be symmetric, i.e.,

$$U_{ab} = U_{ba} \quad \forall a, b \in V, \tag{3}$$

since G does not specify any ordering of the edges;

3. they must be the same for every pair of particles, i.e.,

$$U_{ab} = U \quad \forall a, b \in V, \tag{4}$$

since the edges are not specified with different weights.

For qubit systems, the first condition is met by an *Ising interaction pattern*. For notation convenience, we adopt the controlled phase gate

$$U_{ab}(\phi_{ab}) = e^{-i\phi_{ab}H_{ab}} \quad \text{with} \quad H_{ab} := |1\rangle\langle 1| \otimes |1\rangle\langle 1|, \tag{5}$$

as done in[1], which is an Ising interaction up to rotations on the z-axis at each qubit — since we are interested in entanglement properties of a graph state, we can neglect and omit these rotations (see also[1] for the proof). Finally, since Ising interactions are symmetric, we only need to define $\phi = \phi_{ab} \forall a, b \in V$ to meet all the above constraints. As in[1], we chose $\phi = \pi$ and $|\psi\rangle = \otimes_{a\in V} |+\rangle_a$ so that the resulting state $U_{ab}|\psi\rangle$ is maximally entangled (any reduced state is maximally mixed). This choice also ensures that the gate U_{ab} acts on the corresponding graph creating and deleting the edge $\{a,b\}$ depending if it is contained or not in E. In short, we define a graph state as follows:

Definition 1. *Let $G = (V,E)$ be a graph. The corresponding graph state $|G\rangle$ is given by the following pure state*

$$|G\rangle = \prod_{\{a,b\}\in E} U_{ab} |+\rangle^V, \tag{6}$$

where

$$U_{ab} = \begin{pmatrix} 1 & 0 & 0 & 0 \\ 0 & 1 & 0 & 0 \\ 0 & 0 & 1 & 0 \\ 0 & 0 & 0 & -1 \end{pmatrix}, \tag{7}$$

i.e., a controlled σ_z on qubits a and b.

Physically, it can be pictured as a two-step preparation procedure in which the pure state $|+\rangle$ is prepared at each vertex, and a phase gate U_{ab} is applied to all adjacent vertices a, b in G.

Next, we discuss the graph states used in this paper.

1.2 GHZ states

The N-qubit GHZ state

$$|GHZ\rangle = \frac{|0\rangle^{\otimes N} + |1\rangle^{\otimes N}}{\sqrt{2}} \tag{8}$$

is one of the standard examples of multiparty entangled states. As mentioned previously, these states maximally violate Bell inequalities but are sensitive to loss, and losing any qubit implies destroying all the entanglement content.

The GHZ state corresponds to the star graph and the complete graph Supplementary Fig. S1. This can be seen by applying Hadamard operations to 8 and local complementations to the star graph, which do not change the entanglement content. More specifically, by applying Hadamard operations to all qubits but one (say a) in 8, one obtains a star-graph state with central qubit a, which is equivalent to a complete graph up to local complementation.

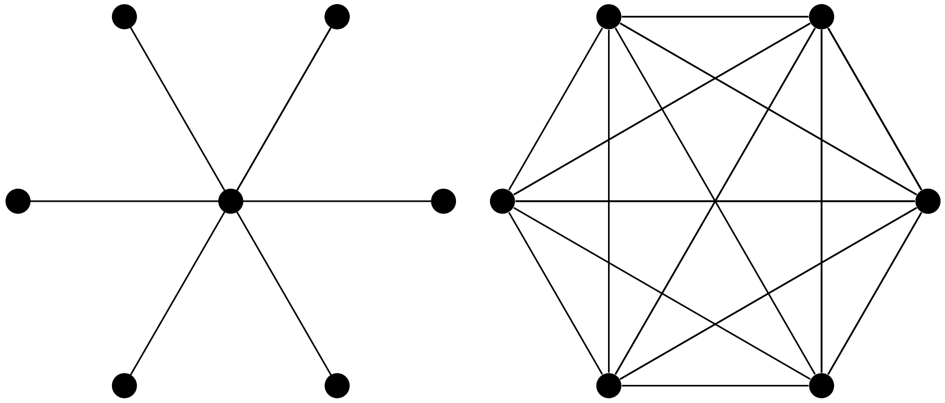

Supplementary Figure S1. Graph representations of the GHZ state: the star (left) and fully connected (right) graphs.

As observed in[2] GHZ states are particularly sensitive (in terms of entanglement content) to loss because their corresponding star graph contains only one central vertex (root). In particular, they showed that all the correlations vanish when a root vertex is lost. This observation leads the Authors to consider redundant roots to build loss-robustness graph states as in the two-centered GHZ graph state depicted in Supplementary Fig. S2 and detailed next.

1.3 Two-centered GHZ graph states

As defined in[2], a two-centered GHZ graph state Supplementary Fig. S2 is a graph state with two root vertices (the graph's centers) connected to one another, and several leaf vertices adjacent to both centers. Such states preserve their entanglement content up to losing some of the qubits, i.e., the remaining graph state still violates a Bell inequality, as carefully assessed in[2]. More precisely, it has been shown[2] that if multiple qubits adjacent to the same root are lost, the remaining state is always a GHZ state, and if qubits adjacent to both roots or the roots themselves are lost, the remaining state is (fully) separable.

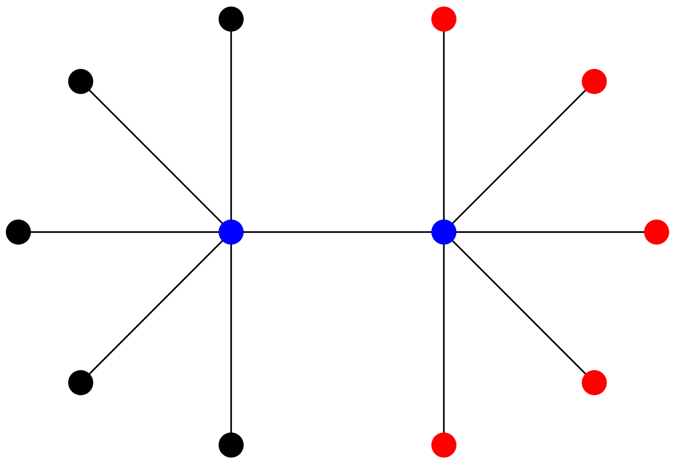

Supplementary Figure S2. The loss-robustness of A two-centered GHZ graph state with 12 qubits. The figure depicts a scenario of loss where the induced graph (obtained by removing the red vertices) is a GHZ state. If any of the two roots (blue vertices) are lost, the induced state is fully separable.

2 Proof of W states' advantage over GHZ in large networks

Here, we present two sufficient conditions so that the quantum state ψ always outperforms the quantum state ϕ in an arbitrarily large lossy network, what we call *ψ's advantage over ϕ*, and show that W and GHZ states satisfy them.

By exploring the monotonically decreasing behavior of

$$\langle E\rangle_{\mathbf{r},\psi}(\varepsilon) := \sum_i q_i(N,\varepsilon)\bar{E}^*_{r_i,\sigma_i}, \tag{9}$$

where

$$\bar{E}^*_{r_i,\sigma_i} := \sup_{\mathscr{L}_i^{r_i}} \sum_{\mathbf{j}} p_{i,\mathbf{j}} E(\rho_{i,\mathbf{j}}), \tag{10}$$

and E is some bipartite entanglement measure, we propose a theorem to identify sufficient conditions in terms of $\langle E\rangle_\psi(\varepsilon)$ and $\langle E\rangle_\phi(\varepsilon)$ so that the N dimensional quantum state ψ outperforms ϕ for any value of loss ε in large networks, i.e., sufficient conditions so that their nontrivial intersection (the threshold), ε_0 s.t. $\langle E\rangle_\psi(\varepsilon_0) = \langle E\rangle_\phi(\varepsilon_0)$ and $\varepsilon_0 \neq 1$, converges to zero when $N \to \infty$. The intuition behind this theorem comes from the following observations.

- If the functions $\langle E\rangle_\psi(\varepsilon)$ and $\langle E\rangle_\phi(\varepsilon)$ have different initial values and both functions monotonically decrease to zero, there must be a non-trivial intersection $\varepsilon_0 < 1$; and
- if $\langle E\rangle_\psi(0) < \langle E\rangle_\phi(0)$ and the $\langle E\rangle_\psi(\varepsilon)$'s derivative with respect to ε, $\langle E\rangle'_\psi(\varepsilon) := \frac{\mathrm{d}\langle E\rangle_\psi(\varepsilon)}{\mathrm{d}\varepsilon}$, decrease faster than $\langle E\rangle'_\phi(\varepsilon)$ for small ε as N increases, then ε_0 converges to zero when $N \to \infty$.

Assuming the first, we formally state:

Theorem 1 (ψ's advantage over ϕ in large lossy networks)**.** *Given two sequences of bounded and monotonically decreasing functions $\{\langle E\rangle_\psi(\varepsilon)\}_N$ and $\{\langle E\rangle_\phi(\varepsilon)\}_N$ if:*

1. $\langle E\rangle_\psi(0) < \langle E\rangle_\phi(0)$ *for all N; and*

2. *$\{\langle E\rangle'_{\phi}(\varepsilon)\}_N$ diverges faster than $\{\langle E\rangle'_{\psi}(\varepsilon)\}_N$ for small ε, e.g.,*

$$\lim_{N\to\infty}\left[\frac{\langle E\rangle'_{\psi}(\varepsilon)}{\langle E\rangle'_{\phi}(\varepsilon)}\right]_{\varepsilon\ll 1}=0 \tag{11}$$

then ε_0 goes to zero when $N\to\infty$.

Here, we prove W's advantage over GHZ states, i.e., we show that W and GHZ states, as ψ and ϕ respectively, satisfy the above theorem.

Proof. Following the above theorem, we need to show that:

1. $\langle\hat{E}\rangle_W(0)<\langle E\rangle_{\text{GHZ}}(0)$ for all N; and
2. $\{\langle E\rangle'_{\text{GHZ}}(\varepsilon)\}_N$ diverges faster than $\{\langle\hat{E}\rangle'_W(\varepsilon)\}_N$ for small ε, e.g.,

$$\lim_{N\to\infty}\left[\frac{\langle\hat{E}\rangle'_W(\varepsilon)}{\langle E\rangle'_{\text{GHZ}}(\varepsilon)}\right]_{\varepsilon=0}=0 \tag{12}$$

We first show the first condition is met. From

$$q_i(N,\varepsilon)=\binom{N-2}{i}\varepsilon^i(1-\varepsilon)^{N-2-i}, \tag{13}$$

we have that $q_0(0,N)=1$ and $q_i(0,N)=0\,\forall i>0$, therefore:

$$\langle\hat{E}\rangle_W(0)=\bar{E}^*_{\sigma_0}<1 \tag{14}$$

and

$$\langle E\rangle_{\text{GHZ}}(0)=\bar{E}^*_{\text{GHZ}}=1, \tag{15}$$

which follows respectively from the fact that W (GHZ) states are probabilistically (deterministically) transformed in a Bell pair[3]. Combining the above equations we find

$$\langle\hat{E}\rangle_W(0)<\langle E\rangle_{\text{GHZ}}(0), \tag{16}$$

as we wanted to prove.

The second condition follows from the loss-robustness of W states[4], i.e.,

$$\lim_{N\to\infty}F(\sigma_0^{N-1},\sigma_1^N)=0, \tag{17}$$

where F is the fidelity. Since only q_i depends on ε, we have

$$\begin{aligned}\langle\hat{E}\rangle'_W(0)&=q'_0(N,0)\bar{E}^*_{\sigma_0^N}+q'_1(N,0)\bar{E}^*_{\sigma_1^N} &(18)\\ &=-(N-2)\left[\bar{E}^*_{\sigma_0^N}-\bar{E}^*_{\sigma_1^N}\right]\\ &=-(N-2)\left[\sup_{\mathscr{L}_i^1}\wp_{0N}E(\sigma_0^N)+\bar{E}^*_{\sigma_0^{N-1}}-\bar{E}^*_{\sigma_1^N}\right], &(19)\end{aligned}$$

where we have used (10) to expand the first term in the second line. Similarly,

$$\langle E\rangle'_{\text{GHZ}}(0)=-(N-2) \tag{20}$$

since $\bar{E}^*_{\sigma_0^N}=1$ and $\bar{E}^*_{\sigma_i^N}=0\,\forall i>0$ in this case. The ratio $\langle\hat{E}\rangle'_W(\varepsilon)/\langle E\rangle'_{\text{GHZ}}(\varepsilon)$ simplifies to

$$\sup_{\mathscr{L}_i^1}\wp_{0N}E(\sigma_0^N)+\bar{E}^*_{\sigma_0^{N-1}}-\bar{E}^*_{\sigma_1^N}. \tag{21}$$

which goes to zero when $N\to\infty$ since $\wp_{0N}$ decreases with N (see Section 2.2.1 in the manuscript) while $E(\sigma_0^N)\in[0,1]$, and $\sigma_0^{N-1}\to\sigma_1^N$ when $N\to\infty$ as assumed in (17).

□

3 Discrete-time finite-state Markov chains

A Markov chain (MC) is a stochastic process defined at integer values of time $r = 0,1,2,\ldots$, that is, for every $r \geq 0$, there is a random variable X_r, the chain state at time r (see also[5]).

Definition 2 (Markov chain process)**.** *The evolution of a MC is defined by* $\{X_r\}_{r\geq 0}$*, where:*

1. *the collection of all possible values of all the* X_r*, the Markovian state space* $\mathscr{X}$*, is a countable set;*
2. *the sampled values of each* X_r *depends only on the most recent (chain) state* X_{r-1}*. More specifically, for all positive r,*

$$P[X_r \mid X_{r-1}, X_{r-2}, \ldots, X_0] = P[X_r \mid X_{r-1}], \tag{22}$$

where the initial (chain) state X_0 *has an arbitrary distribution.*

In such an MC process it is often useful to compute the probability of going to state j in r steps starting in the state i, i.e., $P[X_r = j \mid X_0 = i]$. From the Chapman-Kolmogorov equation, we have that

$$P[X_r = j \mid X_0 = i] = (P^r)_{ij}, \tag{23}$$

where P is the transition probability matrix, whose elements are $P_{ij} = P[X_1 = j \mid X_0 = i]$. That is, $P[X_r = j \mid X_0 = i]$ equals the i,j element of the rth power of matrix P. A finite-state MC is an MC whose Markovian state space is finite. This evolution is often pictorially depicted by nodes (the states) connected by arrows (the transition probabilities).

FSMCs set the mathematical framework to compute $p_{i,\mathbf{j}}$ and to keep track of $\rho_{i,\mathbf{j}}$ in a r-round LOCC transformations $\mathscr{L}_i^r$. More precisely, by associating every set $\{\rho_{i,\mathbf{j}}^r\}_{\mathbf{j}}$ to the sampled values of a random variable X_r for every $r \geq 0$, the process $\{X_r\}_{r\geq 0}$ can be interpreted as an FSMC process — that is, its Markovian state space $\mathscr{X}$ is a finite set, and its evolution depends only on the previous time step.

Proof. The first condition follows directly from the definition

$$p_{i,\mathbf{j}} = \mathrm{Tr}\left(\mathscr{M}_{\mathbf{j}}^{r_i}\right)^{\dagger} \mathscr{M}_{\mathbf{j}}^{r_i} \sigma_i, \tag{24}$$

$$\rho_{i,\mathbf{j}} = \frac{\mathrm{Tr}_{C'} \mathscr{M}_{\mathbf{j}}^{r_i} \sigma_i \left(\mathscr{M}_{\mathbf{j}}^{r_i}\right)^{\dagger}}{p_{i,\mathbf{j}}}, \tag{25}$$

i.e., $\{\rho_{i,\mathbf{j}}^r\}$ corresponds to a finite set of LOCC operations, indexed by $\mathbf{j}$. The second condition is satisfied by definition, i.e., for all $\rho_{i,\mathbf{m}}$ and $\rho_{i,\mathbf{l}} \in \mathscr{X}$ we define the probability of going to state $\rho_{i,\mathbf{m}}$, starting from $\rho_{i,\mathbf{l}}$, as

$$P[X_r = \rho_{i,\mathbf{m}} \mid X_{r-1} = \rho_{i,\mathbf{l}}] := \mathrm{Tr}\, \mathscr{M}_{\mathbf{m}}^1 \mathscr{M}_{\mathbf{m}}^1 \rho_{i,\mathbf{l}}, \tag{26}$$

where $\mathscr{M}_{\mathbf{m}}^1$ is a single-round global measurement given by

$$\left\{\mathscr{M}_{\mathbf{j}}^{r_i} = \bigcirc_{x=1}^{r_i} \mathscr{M}_{j_x}\right\}_{\mathbf{j}=j_{r_i}, j_{r_i-1}, \ldots, j_1}. \tag{27}$$

In other words, (26) corresponds to the probabilities of a single-round LOCC transformation acting on ρ_{il}. □

With this definition,

$$p_{i,\mathbf{j}}^r \equiv P[X_r = \rho_{0\mathbf{j}} \mid X_0 = \sigma_i] = (P^r)_{i\mathbf{j}}, \tag{28}$$

where the last equality follows from (23).

4 Fortescue and Lo's protocol

In[6] Fortescue and Lo present a probabilistic entanglement distillation protocol able to distill Bell pairs from a three-qubit W state. In this protocol, the three parties measure their share of the entangled state, using the set of Kraus operators

$$M_0^{\kappa} = \begin{pmatrix} \sqrt{1-\kappa} & 0 \\ 0 & 1 \end{pmatrix}, \qquad M_1^{\kappa} = \begin{pmatrix} \sqrt{\kappa} & 0 \\ 0 & 0 \end{pmatrix}, \tag{29}$$

if:

1. all parties get outcome 0, they will share the same W state and repeat the protocol;

2. two of the three parties get outcome 0, they will share a Bell pair and successfully terminate the protocol;

3. only one the three parties get outcome 0, they will share a separable state $|0\rangle|0\rangle|0\rangle$ and unsuccessfully terminate the protocol.

These events correspond to repeat, success, and failure events and occur with probability $(1-\kappa)^2$, $2\kappa^2(1-\kappa^2)$ and κ^2. They found that after r executions of the protocol, the maximum average entanglement shared among an unspecified pair of parties is $\frac{r}{1+r}$.

Our protocol is very similar. All parties measure their systems with the same set of Kraus operators (29), but success is deemed not only to Bell states but to any final entangled state, which includes the bipartitions of W states. Moreover, we also apply them to lossy N-party W states $\sigma_{i\neq 0}$.

5 Analytical Expressions for Average Bipartite Entanglement in GHZ, Two-Centered GHZ Graph, and W States

In this section, we provide analytical expressions for our figure of merit (9), for GHZ, two-centered GHZ graph, and W states. We show how to compute (10) and q_i by analyzing the states' resilience to losses.

The received states σ_i of a GHZ transmission are either a GHZ or a completely mixed state, given that none or more than one particle is lost, respectively. In the first case, the optimal LOCC protocol converts a GHZ state into an EPR pair with probability one in a single round, i.e., $\bar{E}^*_{r_0=1,\sigma_0=\mathrm{GHZ}}=1$. In the second case, since $\sigma_{i>0}=\pi_{N-i}$, a classical statistical mixture with no bipartite entanglement, it is impossible to extract any bipartite entanglement from it, and $\bar{E}^*_{r_i,\sigma_{i>0}=\pi_{N-i}}=0$. With these results in hand, only the first term in (9) will contribute, i.e.,

$$\langle E\rangle_{r,\mathrm{GHZ}}(\varepsilon)=\langle E\rangle_{1,\mathrm{GHZ}}=q_0(N,\varepsilon)=(1-\varepsilon)^{N-2}. \tag{30}$$

A similar approach applies for two-centered GHZ graph states, i.e., the optimal LOCC protocols converting the lossy states σ_i are deterministic and known. As before, the received states of a two-centered GHZ graph state transmission are either a GHZ or a completely mixed state, but now with better probabilities; a completely mixed state is only obtained whenever a root qubit is lost (see Section 1 of appendix for further details). Conversely, a GHZ state is obtained with probability

$$q'(N,\varepsilon)=(1-\varepsilon)^N+2\sum_{i=1}^{i_m}\binom{i_m}{i}\varepsilon^i(1-\varepsilon)^{N-i}, \tag{31}$$

where the maximum number of particles that can be lost (still resulting in a GHZ state) is $i_m=\frac{N-2}{2}$, and is deterministically converted to an EPR pair, i.e., $\bar{E}^*_{r_i=1,\sigma_i=\mathrm{GHZ}}=1$. As before, no bipartite entanglement can be extracted from completely mixed states, $\bar{E}^*_{r_i,\sigma_i=\pi_{N-i}}=0$. Therefore, (9) simplifies to

$$\langle E\rangle_{r,\mathrm{GHZ}}(\varepsilon)=\langle E\rangle_{1,\mathrm{GHZ}}=(1-\varepsilon)^N+2\sum_{i=1}^{i_m}\binom{i_m}{i}\varepsilon^i(1-\varepsilon)^{N-i}. \tag{32}$$

For W states, the situation changes dramatically: since optimal LOCC protocols are unknown, there are no closed analytical expressions for (9). As discussed previously, this can be alleviated by assuming the lower bound

$$\langle\hat{E}\rangle_{r,\psi}(\varepsilon):=\sum_i q_i(N,\varepsilon)\sup_\kappa\sum_{\mathbf{j}}p_{i,\mathbf{j}}E(\rho_{i,\mathbf{j}}), \tag{33}$$

which could, in principle, be further manipulated to result in a closed analytical expression. In this work, we have not done this. Instead, we sought for $\sup_\kappa\sum_{\mathbf{j}}p_{i,\mathbf{j}}E(\rho_{i,\mathbf{j}})$ by numerically maximizing $p_{i,\mathbf{j}}E(\rho_{i,\mathbf{j}})$ over a range of values of κ.

References

1. Hein, M. *et al.* Entanglement in graph states and its applications. In *Quantum computers, algorithms and chaos*, 115–218 (IOS Press, 2006).

2. Silberstein, S. & Arnon-Friedman, R. Robustness of Bell violation of graph states to qubit loss. *Phys. Rev. Res.* **5**, 043099, DOI: 10.1103/PhysRevResearch.5.043099 (2023).

3. Chitambar, E., Cui, W. & Lo, H.-K. Entanglement monotones for W-type states. *Phys. Rev. A* **85**, 062316, DOI: 10.1103/PhysRevA.85.062316 (2012).

4. Dür, W. Multipartite entanglement that is robust against disposal of particles. *Phys. Rev. A* **63**, 020303, DOI: 10.1103/PhysRevA.63.020303 (2001).

5. Sericola, B. *Markov chains: theory and applications* (John Wiley & Sons, 2013).

6. Fortescue, B. & Lo, H.-K. Random bipartite entanglement from W and W-like states. *Phys. Rev. Lett.* **98**, 260501 (2007).
